# 3D deep learning versus the current methods for predicting tumor invasiveness of lung adenocarcinoma based on high-resolution computed tomography images

**DOI:** 10.3389/fonc.2022.995870

**Published:** 2022-10-21

**Authors:** Yilv Lv, Ying Wei, Kuan Xu, Xiaobin Zhang, Rong Hua, Jia Huang, Min Li, Cui Tang, Long Yang, Bingchun Liu, Yonggang Yuan, Siwen Li, Yaozong Gao, Xianjie Zhang, Yifan Wu, Yuchen Han, Zhanxian Shang, Hong Yu, Yiqiang Zhan, Feng Shi, Bo Ye

**Affiliations:** ^1^Department of Thoracic Surgery, Shanghai Chest Hospital, Shanghai Jiao Tong University, Shanghai, China; ^2^Department of Research and Development, Shanghai United Imaging Intelligence Co., Ltd., Shanghai, China; ^3^Department of Oncologic Surgery, Shanghai Chest Hospital, Shanghai Jiao Tong University, Shanghai, China; ^4^Department of Radiology, Shanghai Municipal Hospital of Traditional Chinese Medicine, Shanghai University of Traditional Chinese Medicine, Shanghai, China; ^5^Department of Radiology, Yangpu Hospital, Tongji University, Shanghai, China; ^6^Department of Thoracic Surgery, Affiliated Hospital of Gansu Medical College, Pingliang, China; ^7^Department of Thoracic Surgery, Weifang People’s Hospital, Weifang, China; ^8^Department of Thoracic Surgery, Qilu Hospital of Shandong University, Qingdao, China; ^9^Department of Thoracic Surgery, Qingyuan People’s Hospital, Guangzhou Medical University, Guangzhou, China; ^10^Department of Pathology, Shanghai Chest Hospital, Shanghai Jiao Tong University, Shanghai, China; ^11^Department of Radiology, Shanghai Chest Hospital, Shanghai Jiao Tong University, Shanghai, China

**Keywords:** computer-aided diagnosis, lung adenocarcinoma, intraoperative frozen section, tumor invasiveness, artificial intelligence, non-small cell lung (NSCLC)

## Abstract

**Background:**

Different pathological subtypes of lung adenocarcinoma lead to different treatment decisions and prognoses, and it is clinically important to distinguish invasive lung adenocarcinoma from preinvasive adenocarcinoma (adenocarcinoma *in situ* and minimally invasive adenocarcinoma). This study aims to investigate the performance of the deep learning approach based on high-resolution computed tomography (HRCT) images in the classification of tumor invasiveness and compare it with the performances of currently available approaches.

**Methods:**

In this study, we used a deep learning approach based on 3D conventional networks to automatically predict the invasiveness of pulmonary nodules. A total of 901 early-stage non-small cell lung cancer patients who underwent surgical treatment at Shanghai Chest Hospital between November 2015 and March 2017 were retrospectively included and randomly assigned to a training set (n=814) or testing set 1 (n=87). We subsequently included 116 patients who underwent surgical treatment and intraoperative frozen section between April 2019 and January 2020 to form testing set 2. We compared the performance of our deep learning approach in predicting tumor invasiveness with that of intraoperative frozen section analysis and human experts (radiologists and surgeons).

**Results:**

The deep learning approach yielded an area under the receiver operating characteristic curve (AUC) of 0.946 for distinguishing preinvasive adenocarcinoma from invasive lung adenocarcinoma in the testing set 1, which is significantly higher than the AUCs of human experts (P<0.05). In testing set 2, the deep learning approach distinguished invasive adenocarcinoma from preinvasive adenocarcinoma with an AUC of 0.862, which is higher than that of frozen section analysis (0.755, P=0.043), senior thoracic surgeons (0.720, P=0.006), radiologists (0.766, P>0.05) and junior thoracic surgeons (0.768, P>0.05).

**Conclusions:**

We developed a deep learning model that achieved comparable performance to intraoperative frozen section analysis in determining tumor invasiveness. The proposed method may contribute to clinical decisions related to the extent of surgical resection.

## 1 Introduction

Lung cancer ranks second in the most commonly diagnosed cancer and remains the leading cause of cancer death worldwide (1, 2). With the widespread implementation of low-dose computed tomography (CT) screening and regular physical examinations, a substantial number of early-stage lung cancers have been detected (3). Surgical resection remains the gold standard for early-stage lung cancer treatment, and the mode of surgery is lobectomy (4). However, an increasing number of studies and single-institution trials have demonstrated that sublobar resection may yield comparable outcomes in selected patients with early-stage non-small cell lung cancer (NSCLC) (5, 6). Sublobar resection can preserve the lung parenchyma, which is particularly valuable for patients with poor pulmonary reserve or those who are likely to require subsequent additional resection (5). Therefore, sublobar resection is extremely important in the treatment of patients with early-stage NSCLC.

A consistent method has not been established to identify the optimal candidates for sublobar resection of NSCLC with a low likelihood of recurrence. Patients with ground-glass opacity-dominant clinical stage IA adenocarcinomas are suitable for sublobar resection, as confirmed by the latest clinical trial (7). In the new multidisciplinary classification of pulmonary adenocarcinoma by the International Association for the Study of Lung Cancer (IASLC)/American Thoracic Society (ATS)/European Respiratory Society (ERS), the disease-specific survival for adenocarcinoma *in situ* (AIS) and minimally invasive adenocarcinoma (MIA) are 100% or nearly 100%, respectively, after complete resection. Invasive lung adenocarcinoma (IAC) is more aggressive and has a worse prognosis than AIS and MIA, suggesting that sublobar resection is only appropriate for patients with MIA or AIS (8, 9).

Currently, there are three methods to evaluate pathological aggressiveness and the suitability of sublobar resection in patients with early-stage lung adenocarcinoma: preoperative biopsy, CT imaging, and intraoperative frozen section analysis. Small lesions are difficult to locate, while biopsy samples may not be representative (10, 11). In addition, whether preoperative biopsy increases the likelihood of early-stage lung cancer recurrence remains controversial (12, 13). Intraoperative frozen section analysis has traditionally been used to assess tumor invasiveness and guide surgical management. However, the technique does have certain limitations: Several studies have shown that the accuracy and sensitivity of intraoperative frozen sections are relatively low for subcentimeter pulmonary nodules (14, 15). There has been a strong focus on identifying pathological invasiveness according to imaging findings. CT imaging can reportedly distinguish preinvasive lung adenocarcinoma (pre-IAC; AIS and MIA) from IAC, although the small sample sizes and ambiguous appearances of these findings prevent its routine adoption in clinical practice (16–20). It is therefore a great challenge for radiologists or experts to diagnose a large number of detected pulmonary nodules, as these methods are time-consuming and error-prone when interpreting nodules. Therefore, we need a more straightforward and precise method to determine the pathological aggressiveness of all types of nodules based on CT imaging, not just ground-glass nodules.

In recent years, artificial intelligence (AI) techniques coupled with radiological imaging have played an essential role in automatically predicting the tumor invasiveness of pulmonary adenocarcinomas from CT scans (21–25). Deep learning, a popular research area of AI, enables end-to-end models to obtain self-learned features and achieves promising results using input data without the need for manual feature extraction (26). Deep learning algorithms have been widely applied to many problems, such as lung nodule detection, segmentation, and classification (27, 28).

The purpose of this study was to develop a computer-aided approach to accurately and automatically discriminate the invasiveness of lung adenocarcinomas in routine chest CT images. We built a deep learning model and investigated the utility of the model in predicting pathological invasiveness among patients with early-stage lung adenocarcinoma. In addition, we compared the performance of the deep learning model with that of observers and intraoperative frozen section diagnoses to determine the best method of distinguishing pre-IAC from IAC in clinical practice.

## 2 Methods

### 2.1 Ethical considerations

This retrospective study adhered to the Declaration of Helsinki and relevant ethical policies in China. The study protocol was approved by the Institutional Review Board and Ethics Committee of Shanghai Chest Hospital (No. IS2180). The requirement for patient consent was waived because of the retrospective study design.

### 2.2 Data collection

**Figure 1:**
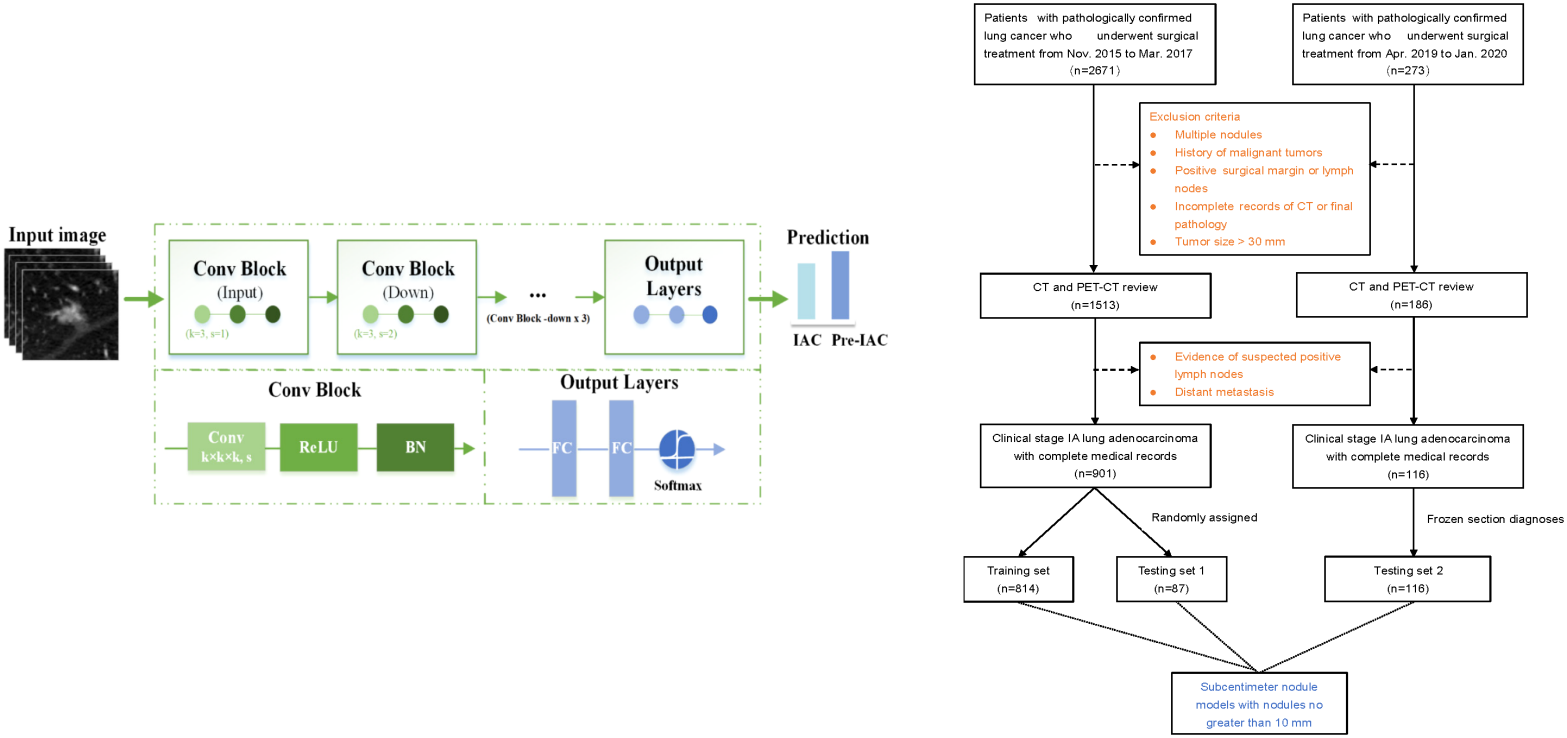
The flow chart of patient selection and deep learning architecture. “Conv” represents a convolution, “k” represents the kernel, and “s” denotes the number of strides. “BN” represents the batch normalization layer. “FC” represents a fully connected layer.

This study retrospectively reviewed the medical records of 2671 consecutive patients with NSCLC who underwent surgical resection in Shanghai Chest Hospital between November 2015 and March 2017 to develop the training set and testing set 1. An additional dataset of 273 patients who underwent surgery between April 2019 and January 2020 was separately identified and formed an additional testing set (i.e., testing set 2). The inclusion criteria were as follows: (1) stage 0 or IA lung adenocarcinoma confirmed by final pathology according to the 8th Edition of the TMN Classification (29); (2) availability of preoperative thin-section CT (0.625 mm–1.25 mm) images; and (3) resected nodules were sent for paraffin sectioning, and the final pathological results were available. The exclusion criteria were as follows: (1) multiple pulmonary nodules; (2) previous history of malignant tumor; (3) pathologically confirmed positive surgical margin or lymph nodes; (4) incomplete records of CT or pathology quality and (5) pulmonary nodule with size greater than 30mm. Finally, 901 patients with early-stage lung adenocarcinoma were enrolled and testing set 1 using a stratified random sampling method, and 116 patients were enrolled in the testing set 2. To compare the accuracy of intraoperative frozen section analysis with that of artificial intelligence-based CT image analysis, frozen section diagnoses of the independent testing set 2 were collected (**Figure 1**).

### 2.3 CT image acquisition, classification, and pathological evaluation

All preoperative CT scans were obtained at full inspiration to avoid respiratory motion artifacts. Brilliance iCT and Ingenuity (Philips Medical Systems, Netherlands) scanners were used to scan CT images at an efficient dose of 120 kV tube energy and 200 mA. All CT data were acquired in the supine position at full inspiration. High-resolution images were acquired with a reconstruction slice thickness of 1 mm and no overlap, and a lung window (window width: 1500, window level: -500) was used for film reading.

**Figure 2:**
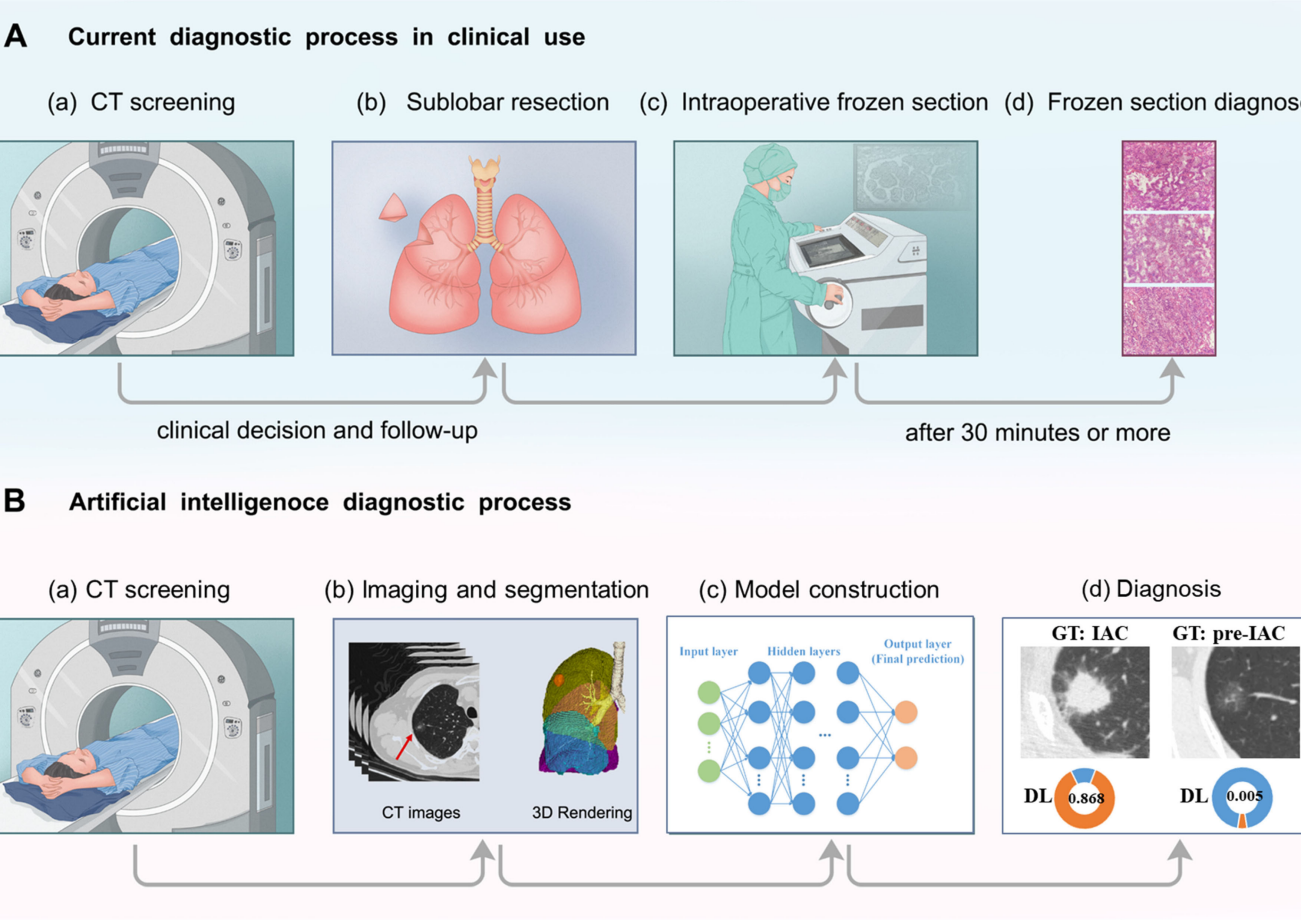
Diagram of **(A)** current and **(B)** artificial intelligence procedures to determine histological invasiveness. In the current diagnostic process in clinical use, sublobar resection is performed and intraoperative frozen sections decide the extent of surgery. In the other hand, in the workflow of deep learning approach, extensive information could be extracted from CT images, and help with the determination of tumor invasiveness. “GT” refers to ground truth. “DL” represents deep learing.

For frozen section diagnosis, resected tumor tissues were preserved in a sterile, sealed plastic bag; they were sent to the pathology department within 5 min after resection. Essential tumor information was recorded; one block of the largest tumor tissue was separated from the sample and sectioned using a CM-3050s freezing microtome (Leica, Nussloch, Germany). Before sectioning, the tissue block was frozen at -24°C for 5 min in OCT compound (SAKURA Tissue-Tek; Torrance, CA, USA). One or two slices (5 µm each) were collected and placed on glass slides. The slides were fixed in methanol/glacial acetic acid for 10–20 s and then subjected to routine hematoxylin and eosin staining (**Figure 2**). The predominant pattern was defined according to the histologic component with the greatest percentage.

For paraffin-embedded sections, any remaining tissues that had been collected during surgery were fixed in 10% formaldehyde, embedded in paraffin, continuously sectioned at 5 μm, and subjected to hematoxylin and eosin staining for postoperative pathological analysis. Final pathology was also established *via* elastic fiber staining and immunohistochemical assessment of cytokeratin 7, thyroid transcription factor-1, and napsin A (all antibodies from Cell Signaling Technology; Danvers, MA, USA) in paraffin-embedded sections.

Frozen section and final pathology diagnoses came from blind assessments by two pathologists (Y.H. and Z.S., chest pathologists with more than 20 years of experience in pathological diagnosis) according to the IASLC/ATS/ERS classification (8). Two pathologists reevaluated the diagnoses to reach a consensus if a discrepancy presented. AIS and MIA were combined to form a low-risk group that was called pre-IAC.

### 2.4 Nodule labeling and segmentation

All lung nodules with nodule diameters greater than 3 mm on each CT scan were automatically localized with 3D bounding boxes and automatically segmented using a research tool (30) developed by Shanghai United Imaging Intelligence Co., Ltd. A total of 1017 nodules were ultimately included as regions of interest (ROIs), and each of them was reviewed and confirmed by at least two senior radiologists. **Supplementary Material Figure S1** illustrates the size distribution of pre-IAC and IAC nodules on diameter.

### 2.5 Deep learning model construction

In the data preprocessing step, we first used the lung window (window width: 1500, window level: -400) for CT images normalization by Z-score standardization method. Then we truncated the normalized intensity value into the range of [-1,1], which means the values below -1 would be set to -1, and the values above 1 would be set to 1. The whole equation is defined as follows (31, 32).


$$I=\{-1,if\;\frac{I-mean}{STD}<-11,if\;\frac{I-mean}{STD}>1\frac{I-mean}{STD},other$$

Where *I* refers to the CT intensity value, *mean* is the window level of -400, and the *STD* is set as the half of window width of 1500.

Before feeding the images into the deep learning network, we resampled each of the CT image to a spacing of 0.2×0.2×1.0 mm (3), extracted the nodule in a bounding box, and then resized the nodule bounding box to a 3D path with size of 144×144×32 pixels. Note that the bounding box was expanded by 20% to include more surrounding lung parenchyma information. In this way, the small nodules could be enlarged instead of occupying only a small region in the patch. Similarly, large nodules could be shrinked so that the box could include the whole nodule. To avoid overfitting and increase the robustness of the deep learning network, image augmentation, including rotation, scaling, and flipping, was performed on each image with a probability of 0.5. Rotation was randomly performed with an angle along an axis in a range of −5° to 5°. The scaling factor was randomly sampled in a range from 0.75 to 1.25. Flipping was adopted randomly along each axis.

The deep learning model was built by using a convolutional neural network (CNN), consisting of one input block, four downsample blocks, and one output block (**Figure 1**). A 3D convolution layer with a 3×3×3 kernel filter is used as the input block. The downsample block consists four 3D convolution layers, each with 3×3×3 filters and a stride of 2, followed by a batch normalization and a rectified linear unit (ReLU) layer, respectively. After that, the output block consists two fully connected layers followed by a ReLU layer and a softmax function to make a decision by providing the predicted probabilities for pre-IAC and IAC.

The proposed model was implemented using Python (version = 3.7.0) based on the platform of PyTorch (version = 1.7.0), and experiments were performed on a workstation with NVIDIA Quadro RTX 6000 24GB GPU and Intel(R) Xeon(R) Gold 6230R CPU. Adam was used as optimizer for stochastic gradient descent with an initial learning rate of 10^-4^, weight decay of 0.01 and a batch size of 64 to update the network. The learning rate is halved if the validation performances do not improve during 100 epochs. To avoid potential overfitting, we used an early stop when the learning rate drops below 10^-6^ or 1000 epochs were exceeded. Focal loss function was applied (33, 34). Note that the deep learning model used only the image information where clinical features were not included.

### 2.6 Subcentimeter nodule classification model construction

Considering that small nodules are more difficult to discriminate than nodules with larger sizes, we collected subcentimeter nodules with sizes no greater than 10 mm from the training set, testing set 1, and testing set 2. We then trained a specific model on the subcentimeter nodules of the training set, with the same training strategies used for deep learning model construction. The performance was evaluated on the testing set 1 and testing set 2 (**Figure 1**).

### 2.7 Observer study

For human performance comparisons, two radiologists, two junior surgeons, and two senior surgeons were recruited. They were blinded to the clinical records and pathological results and diagnosed all the nodules with only CT images. Each reader read the CT images independently and classified the nodules into pre-IAC or IAC, as with the deep learning model.

### 2.8 Statistical analysis

Age, sex, smoking history, surgical procedure, tumor size, and location of each patient were analyzed. Pearson’s χ2 test or Fisher’s exact test was used to compare frequencies of categorical variables (all continuous variables were converted to categorical variables except for age, as shown in **Table 1**). The Mann-Whitney U test was used to analyze the age between the two groups. The diagnostic performance of artificial intelligence models, observers, and frozen section diagnoses was evaluated by the area under the receiver operating characteristic (ROC) curve (AUC) and other evaluation metrics, such as accuracy, sensitivity, specificity, and Matthews correlation coefficient (MCC). The DeLong test was performed to compare the AUC curves of the deep learning models and observer studies and intraoperative frozen section, and the 95% confidence interval (95% CI) of the AUC was also assessed. In addition, the statistical significance of the difference in accuracy between deep learning models, observers, and frozen section diagnoses was evaluated using Pearson’s χ2. All statistical analyses reported in this study were performed with Python (Version 3.7.0) and R (Version 4.0.2), and a P value less than 0.05 was considered statistically significant.

**Table 1:** Clinicopathologic characteristics of the patients in the main set (including the training set and testing set 1) and testing set 2.

Characteristic	Main set			Testing set 2		
	AIS/MIA	IAC	P-value	AIS/MIA	IAC	P-value
Mean age	52	59	<0.001	53	61	<0.001
Sex			<0.001			0.88
Female	276	302		32	46	
Male	98	225		16	22	
Smoking history			<0.001			0.078
Yes	193	376		14	29	
No	181	151		34	39	
Diameter (cm)			<0.001			<0.001
≤1.0	301	50		38	16	
1.0–2.0	72	343		9	41	
≥2.0	1	134		1	11	
Nodule type Solid nodule Subsolid nodule	35 339	270 257	<0.001	0 48	28 40	<0.001
Location			0.856			0.544
RUL	128	182		17	17	
RML	30	36		4	4	
RLL	69	102		8	17	
LUL	106	140		16	22	
LLL	41	67		3	8	
Surgical type			<0.001			<0.001
Wedge resection	137	39		21	9	
Segmentectomy	84	56		10	9	
Lobectomy	153	432		17	50	

AIS, adenocarcinoma in situ; MIA, minimally invasive adenocarcinoma; IA, invasive adenocarcinoma; RUL, right upper lobe; RML, right middle lobe; RLL, right lower lobe; LUL, left upper lobe; LLL, left lower lobe. p-values were calculated using t-tests and Pearson’s chi-squared tests.

## 3 Results

### 3.1 Clinicopathological characteristics of all nodules in pre-IAC group and IAC group

A total of 1017 nodules (pre-IAC/IAC: 422/595) were included. The clinicopathological characteristics are summarized in **Table 1**. Significant differences were found in terms of age, sex, smoking history, nodule diameter, and surgical type in the main set (P< 0.05). There were also significant differences between AIS/MIA and IAC in terms of age, nodule diameter, and surgical type in the testing set 2 (P< 0.05). Detailed information of the nodules for the overall and subcentimeter nodule classification is provided in **Supplementary Material Table S1**.

### 3.2 Evaluation of classification performance on all nodules

#### 3.2.1 Deep learning model

The deep learning model was trained on 540 epochs, and after convergence, the weights were used for testing. In **Table 2**, the results show that the deep learning model achieved an AUC of 97.9% (95% CI: 96.8-99.0) with a sensitivity of 91.8%, specificity of 91.5% and accuracy of 91.6% on the training set, and AUC of 0.946 (95% CI: 89.9–99.4) with a sensitivity of 86.5%, specificity of 91.4%, and accuracy of 88.5% on the testing set 1. The AUC, sensitivity, specificity and accuracy on testing set 2 are 0.862 (95% CI: 79.4–93.0), 73.5%, 91.7%, and 81.0%, respectively. Note that the testing set 1 was acquired in the same time period with training set (2015-2017), while the testing set 2 was collected 4 years later (2019-2020). This may contribute the slightly reduced performance in testing set 2. The distribution differences of the deep features between the main set and testing set 2 were illustrated in **Supplementary Material Figure S2**.

**Table 2:** The performance of overall nodules with various methods for predicting pathological invasiveness.

	F1-score	Sensitivity	Specificity	MCC	ACC	AUC
Training set						
	0.928	0.918	0.915	0.829	0.916	0.979 [0.968,0.990]
Testing set 1						
Deep Learning Model	0.900	0.865	0.914	0.769	0.885	0.946 [0.899,0.994]
Radiologists^*^	0.869	0.913	0.714	0.666	0.833	0.809 [0.711,0.907]
Thoracic Surgeons (Junior)^*^	0.813	0.724	0.913	0.624	0.799	0.823 [0.728,0.918]
Thoracic Surgeons (Senior)^*#^	0.740	0.615	0.929	0.546	0.741	0.779 [0.675,0.883]
Testing set 2						
Deep Learning Model	0.820	0.735	0.917	0.644	0.810	0.862 [0.794,0.930]
Radiologists	0.818	0.829	0.720	0.558	0.784	0.776 [0.687,0.865]
Thoracic Surgeons (Junior)	0.759	0.662	0.885	0.544	0.754	0.768 [0.678,0.858]
Thoracic Surgeons (Senior)^*^	0.675	0.551	0.896	0.463	0.694	0.720 [0.623,0.817]
Frozen Section^*^	0.820	0.676	0.833	0.624	0.741	0.755 [0.663,0.847]

^*^Significant difference found between this diagnostic method of AUC and deep learning model by the DeLong test (P<0.05).

^#^Significant difference found between this diagnostic method of accuracy and the deep learning model by Pearson’s χ2 test (P<0.05).

#### 3.2.2 Observer study with radiologists and surgeons

For the results of testing set 1, the two radiologists achieved the highest averaged accuracy of 83.3% and AUC of 0.809 (95% CI: 71.1–90.7), the two junior thoracic surgeons obtained a mean accuracy of 79.9% and AUC of 0.823 (95% CI: 72.8–91.8), and the two senior thoracic surgeons achieved a mean accuracy of 74.1% and AUC of 0.799 (95 CI: 67.5-85.8). All of the averaged AUC of the observer studies were significantly lower than that of the deep learning model by the DeLong test (*P*< 0.05). Significantly decreased accuracy was found in the assessment of senior thoracic surgeons than that of deep learning with Pearson’s χ2 test.

For the testing set 2, the mean accuracy of radiologists, junior thoracic surgeons, and senior thoracic surgeons is 78.4%, 75.4%, and 69.4%, separately, meanwhile, the averaged AUC of the three observer studies is 0.776 (95 CI: 68.7-86.5), 0.768 (95 CI: 67.8-85.8) and 0.720 (95 CI: 66.3-84.7), respectively. Significantly decreased AUC was only found in the senior thoracic surgeons’ assessment than that of the deep learning model (DeLong test, P<0.05). Detailed mean AUC, accuracy, sensitivity, specificity, MCC, and F1-score of the six observers are shown in **Table 2**.

#### 3.2.3 Intraoperative frozen section analysis

**Figure 3:**
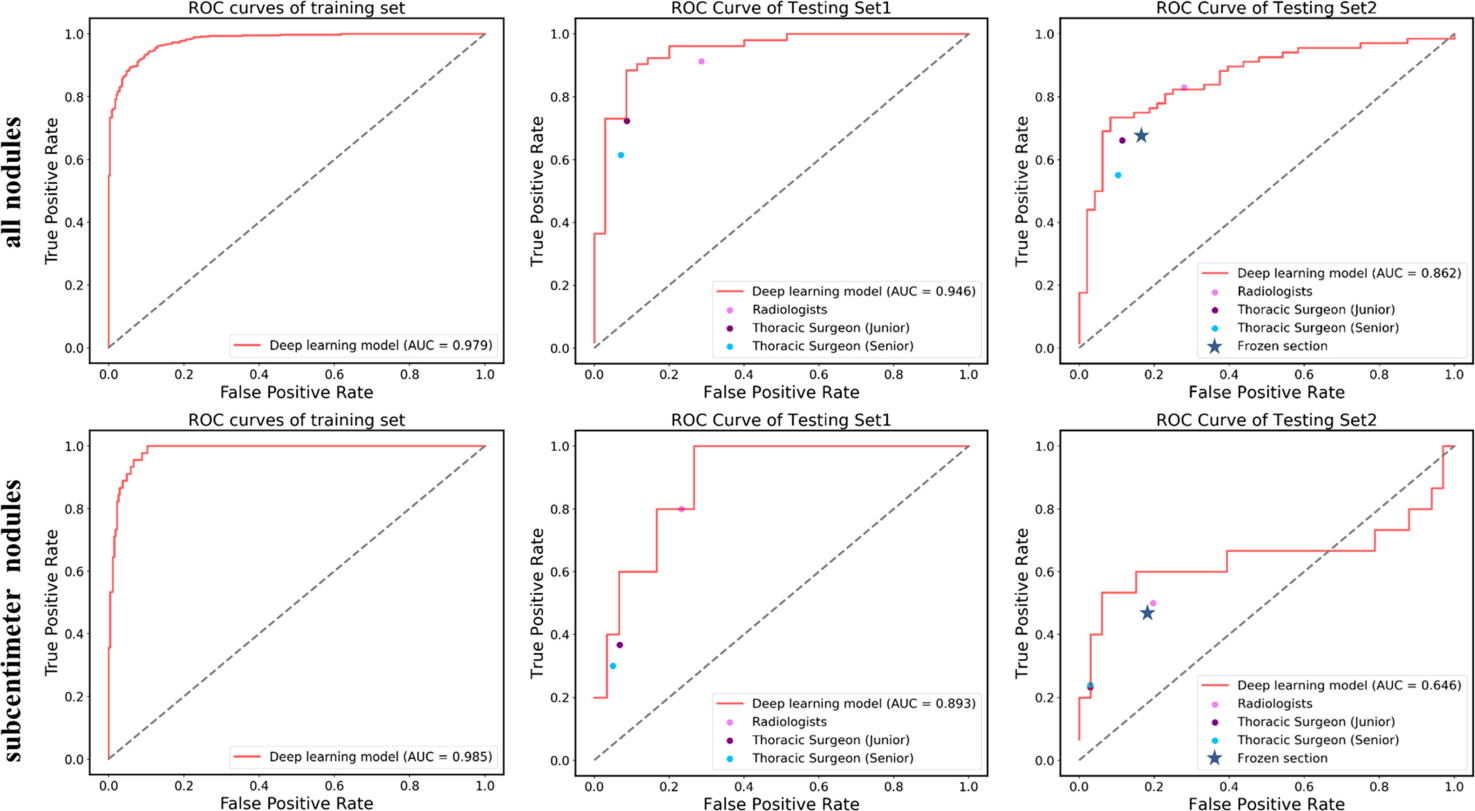
ROC curves showing the performance of the deep learning model and current methods in distinguishing pre-IAC from IAC in testing set 1 and testing set 2. Note that the results of frozen sections as well as radiologists and surgeons do not have probabilities and they were shown as line or dots in the figure.

Due to the availability, in this study, intraoperative frozen section diagnosis was analyzed in the testing set 2 for distinguishing pre-IAC from IAC in clinical practice. The accuracy of frozen sections for overall nodules was 74.1%, which was lower than that of the deep learning approach (81.0%) (**Table 2**). Intraoperative frozen section analysis yielded AUC values of 0.755 (95% CI: 66.3–84.7). Compared to frozen section analysis, the deep learning approach achieved significantly higher AUC values at 0.862 (P<0.05) (**Figure 3**).

#### 3.2.4 Evaluation of classification performance on nodules with subcentimeter size

Nodules with subcentimeter size refer to the nodules with sizes no greater than 10 mm. In comparison to large nodules, they are more difficult to be differentiated between pre-IAC and IAC due to their small size. Considering that, we particularly repeated the above experiments for these subcentimeter size nodules.

As shown in **Table 3**, the deep learning model achieved a sensitivity of 95.6%, specificity of 93.4%, accuracy of 93.7%, and AUC of 98.5% (95% CI: 97.3–99.6) on the training set, and a sensitivity of 60.0%, specificity of 90.0%, accuracy of 85.7%, and AUC of 89.3% (95% CI: 77.2–100.0) on the testing set 1. In testing set 2, the deep learning model achieved a sensitivity of 40.0%, specificity of 97.0%, accuracy of 85.7%, and AUC of 0.646 (95% CI: 42.9–86.4).

**Table 3:** The performance of subcentimeter nodules (<10 mm) with various methods for predicting pathological invasiveness.

	F1-score	Sensitivity	Specificity	MCC	ACC	AUC
Training set						
	0.811	0.956	0.934	0.787	0.937	0.985 [0.973,0.996]
Testing set 1						
Deep Learning Model	0.545	0.600	0.900	0.464	0.857	0.893 [0.772,1.00]
Radiologists	0.554	0.800	0.767	0.481	0.771	0.783 [0.597,0.969]
Thoracic Surgeons (Junior)	0.422	0.367	0.932	0.340	0.843	0.667 [0.430,0.904]
Thoracic Surgeons (Senior)	0.367	0.300	0.950	0.360	0.857	0.683 [0.452,0.914]
Testing set 2						
Deep Learning Model	0.545	0.400	0.970	0.486	0.857	0.646 [0.429,0.864]
Radiologists	0.515	0.500	0.803	0.324	0.708	0.661 [0.422,0.900]
Thoracic Surgeons (Junior)	0.349	0.233	0.970	0.311	0.740	0.585 [0.324,0.846]
Thoracic Surgeons (Senior)	0.345	0.239	0.970	0.334	0.740	0.585 [0.324,0.846]
Frozen Section	0.500	0.467	0.818	0.297	0.708	0.642 [0.397,0.887]

Note that no significant difference was found between the AUC curves by the DeLong test (P>0.05), and no significant difference was found between the accuracies by Pearson’s χ2 test (P>0.05).

For subcentimeter nodules, deep learning models also yielded higher accuracies than the six observers (**Table 3**). Notably, the mean sensitivities of the two radiologists were higher than those of the artificial intelligence models in both testing set 1 and testing set 2, at 80.0% and 50.0%, respectively.

Likewise, the accuracy of frozen sections for subcentimeter nodules was 70.8%, lower than the accuracy of the artificial intelligence model (**Table 3**). Intraoperative frozen section analysis yielded AUC values of 0.642 (95% CI: 39.7–88.7) for subcentimeter nodules, which is lower than that of the deep learning approach, at 0.646 (P>0.05) (**Figure 3**).

## 4 Discussion

Accurately discriminating pre-IAC from IAC is of great value for preoperative clinical guidance since there are significant differences in the 5-year disease-free survival rate between pre-IAC and IAC (9, 35). AI techniques can capture subtle information from CT images and learn a large number of features or deep representations of a given pulmonary nodule without any additional clinical information. AI techniques integrated with medical images have shown advantages in the invasive classification of lung adenocarcinoma (23, 36, 37). For instance, Wang et al. (21) used 886 ground-glass nodules (GGNs) from 794 patients to predict the invasiveness of lung adenocarcinoma using a deep learning network with an AUC of 0.941. In the clinic, the type of lung adenocarcinoma is identified by histological examination (e.g., biopsy and surgical resection), and diagnosis through CT image review is error-prone and time-consuming. In our study, the deep learning model achieved good discrimination on both testing set 1 and testing set 2 in terms of the overall nodule size (with AUCs of 0.946 and 0.862, respectively). Although histological examination may still be the gold standard, the method presented in this study provides a convincing, non-invasive method for initial diagnosis before surgical resection.

In this study, the deep learning approach achieved better AUC and accuracy than observers in overall and subcentimeter nodules. The deep learning approach achieved a significantly higher AUC than that of human experts for overall nodules in the testing set 2 (P<0.05). The diagnostic accuracy of well-trained radiologists was slightly lower than that of the deep learning model and higher than the accuracies of thoracic surgeons. Radiologists and surgeons typically focus on visible features such as size, solid components, lesion margin, and other qualitative features, which might be less sensitive to the local evidence that may be exploited by deep learning models. The low accuracy of thoracic surgeons in distinguishing pre-IAC from IAC may relate to the insufficient training and experience of surgeons. Previous studies have reported that deep learning-derived models can achieve equivalent and even higher performance than radiologists; the results of our study support this assertion.

Intraoperative frozen sections are a reliable and routinely used procedure for deciding the extent of surgery (**Figure 2A**). This study shows that the deep learning approach achieved comparable performance to frozen sections in determining tumor invasiveness, which could largely improve the current nodule screening process using CT images. For instance, our deep learning model might provide additional information on suspicious nodules, and doctors could integrate this information with patient history and clinical symptoms to guide the treatment plan. Patients with pre-IAC nodules predicted by a deep learning model might be more suitable for follow-up monitoring, avoiding invasive surgery. In addition, it only takes a few minutes to detect a patient’s lung nodules in CT images based on AI, while intraoperative frozen sections take hours to complete, which can greatly reduce the patient’s waiting time. Furthermore, to our knowledge, comparisons of the diagnostic accuracy of frozen sections and CT-derived deep learning approaches have not yet been reported. Qiu et al. (38) and Wang et al. (39) compared the diagnostic accuracy of CT-based radiomics methods with that of frozen section analysis for the pathological classification of early-stage lung adenocarcinoma. Qiu et al. (38) reported that the AUC of the nomogram was 0.815, and that of the frozen section analysis was 0.670 (P=0.00095). In this study, the AUC of the deep learning approach was 0.862 in the testing set 2 for overall nodules and 0.755 for intraoperative frozen section, which is higher than the study of Qiu et al. (38). The study of Qiu et al. (38) classified AAH, AIS, MIA and lepidic predominant adenocarcinoma (LPA) into pre-IAC because of the high 5-year survival of LPA, which made it more difficult for pathologists to distinguish LPA from other invasive adenocarcinomas in frozen sections. This may have contributed to the lower AUC of frozen sections in their study. The study of Wang et al. (39) reported no significant difference in the overall diagnostic accuracy between the radiomics method and FS (68.8% vs. 70.0%, P = 0.836), which is consistent with the results of our study.

Clinically, many factors affect intraoperative frozen section diagnoses, such as tumor size, sampling issues, and even nodule density. Liu et al. (40) reported that the diagnostic accuracy of FS for tumors smaller than 1 cm and larger than 1 cm in diameter was 79.6% and 90.8%, respectively. Yeh et al. (41) reported an average frozen section diagnostic accuracy of 64% (54% to 74%) for discriminating among AIS, MIA, and invasive adenocarcinomas by five pathologists. In this study, the accuracies of frozen sections for overall and subcentimeter nodules were 74.1% and 70.8%, respectively. Discrepancies were mostly due to the underestimation of AIS and MIA. A high percentage of AIS/MIA and concurrent subcentimeter nodules may be one of the reasons for the high accuracy of the study of Liu et al. (40). Moreover, Zhu et al. (42) analyzed 803 cases and reported that misdiagnosis by frozen sections because of sampling error might lead to incomplete resection. Our study results suggest that a deep learning approach could serve as a reliable and complementary method when pathological evaluation cannot be performed intraoperatively.

However, this study still has several limitations. First, this is a retrospective study conducted at a single institution and is therefore subject to potential biases concerning patient selection, measurements, and observers. Prospective and multicenter trials are required in future studies. Second, intraoperative frozen sections also aid in determining the resection margin, which is not supported yet in the proposed deep learning approach. Therefore, another interesting research direction for the deep learning approach is to estimate appropriate surgery margin in clinical application. Third, efficient integration of the deep learning approach into clinical workflows still needs to be explored. Fourth, the sample size of subcentimeter nodules in the testing set was relatively low, which may decrease the model generalizability. Future work should include a large number of subcentimeter nodules to improve the performance of a deep learning approach in predicting tumor invasiveness.

## 5 Conclusion

We used a deep learning approach that demonstrated plausible performance, and its ability to distinguish tumor invasiveness was comparable to that of intraoperative frozen section analysis. This deep learning approach has potential value in clinically guiding surgical strategies, but it still needs to be verified in prospective and multicenter trials.

## Data availability statement

The raw data supporting the conclusions of this article will be made available by the authors, without undue reservation.

## Ethics statement

The study was conducted in accordance with the Declaration of Helsinki (as revised in 2013). The study was approved by the Institutional Review Board of Shanghai Chest Hospital, and individual consent for this retrospective analysis was waived.

## Author contributions

Conception and design: YL, YZ, BY. Administrative support: YZ, YH, HY. Provision of study materials or patients: BY, YH, HY. Collection and assembly of data: YL, RH, JH, KX, YG, XianZ, YWu. Data analysis and interpretation: YL, YWe, XiaoZ, ML, CT, LY, BL, YH, ZS. All authors contributed to the article and approved the submitted version.

## Funding

This work was supported by the Interdisciplinary Program of Shanghai Jiao Tong University (grant no. YG2014QN22), Cooperative Research Project of Shanghai Jiao Tong University Collaborative Innovation Center of Translational Medicine (TM201822), Shanghai Science and Technology Support Project (No. 19441908900), National Science and Technology Innovation 2030-Major Project (2021ZD0111103), and National Natural Science Foundation of China (82172030, 82001812).

## Acknowledgments

We thank Dr. Zhichao Liu for providing valuable suggestions in the revision of this manuscript.

## Conflict of interest

YWe, YG, XianZ, YWu, YZ and FS are employees of Shanghai United Imaging Intelligence Co., Ltd. The company has no role in performing the surveillance and interpreting the data.

The remaining authors declare that the research was conducted in the absence of any commercial or financial relationships that could be construed as a potential conflict of interest.

## Publisher’s note

All claims expressed in this article are solely those of the authors and do not necessarily represent those of their affiliated organizations, or those of the publisher, the editors and the reviewers. Any product that may be evaluated in this article, or claim that may be made by its manufacturer, is not guaranteed or endorsed by the publisher.

## Abbreviations

AUC, area under the curve; NSCLC, non-small cell lung cancer; IASLC, International Association for the Study of Lung Cancer; ATS, American Thoracic Society; ERS, European Respiratory Society; AIS, adenocarcinoma in situ; MIA, minimally invasive adenocarcinoma; IAC, invasive lung adenocarcinoma; pre-IAC, preinvasive lung adenocarcinoma; AI, artificial intelligence; CNN, convolutional neural network; LPA, lepidic predominant adenocarcinoma.
