# Supplementary material for: 3D deep learning versus the current methods for predicting tumor invasiveness of lung adenocarcinoma based on high-resolution computed tomography images
Source: Front Oncol. 2022 Oct 21;12:995870. doi: 10.3389/fonc.2022.995870 (PMC9634256; doi:10.3389/fonc.2022.995870)
Supplement: Supplementary file 1 [file DataSheet_1.docx]

**Table S1.** Numbers of overall and subcentimeter pulmonary nodules for the training set, testing set 1 and testing set 2

|  | Overall nodules | | | | Subcentimeter nodules (<10 mm) | | | |
| --- | --- | --- | --- | --- | --- | --- | --- | --- |
| Final Pathology | Training set | Testing set 1 | Testing set 2 | Total | Training Set | Testing set 1 | Testing set 2 | Total |
| AIS/MIA | 339 | 35 | 48 | 422 | 272 | 30 | 33 | 335 |
| IAC | 475 | 52 | 68 | 595 | 45 | 5 | 15 | 65 |
| Total | 814 | 87 | 116 | 1017 | 317 | 35 | 48 | 400 |

Abbreviations: AIS = adenocarcinoma in situ, MIA = minimally invasive adenocarcinoma, IAC = invasive lung adenocarcinoma.

**Illustration of size distribution of pre-IAC and IAC nodules on diameter**

In this study, 73.5% (310 nodules) of pre-IAC were between [5, 10) mm, while 64.0% (381 nodules) of IAC were between [10, 20) mm.

**
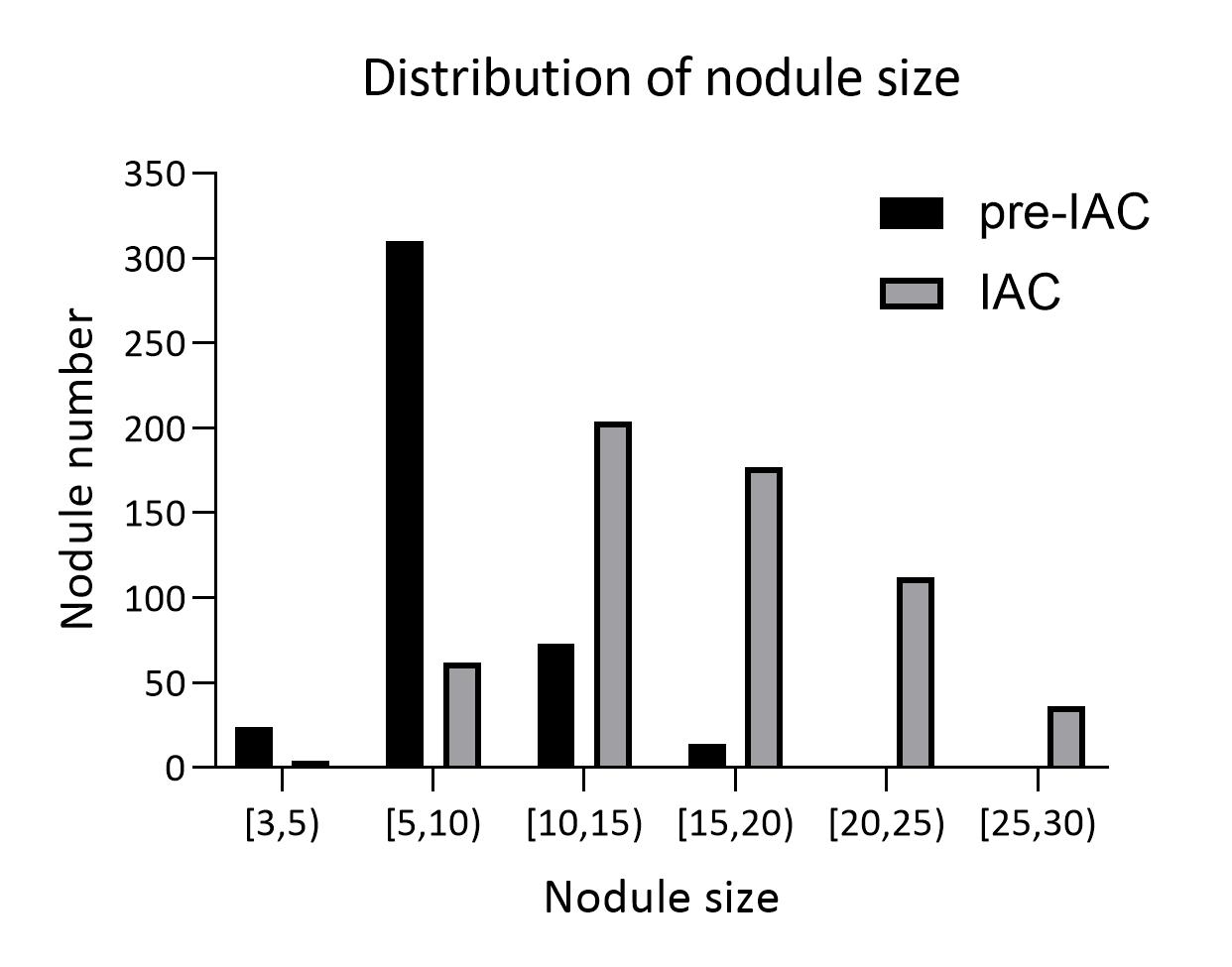
**

**Figure S1**. Size distribution of pre-IAC and IAC nodules on diameter. Pre-IAC includes adenocarcinoma in situ (AIS) and minimally invasive adenocarcinoma (MIA). IAC = invasive lung adenocarcinoma.

**Comparison of deep features between the main set and testing set 2**

We used t-distributed stochastic neighbor embedding (t-SNE)^1^ to display the feature distributions of the two groups. A total of 256 deep features extracted from the output of the first fully connected layer were utilized as input of t-SNE and projected into 2 dimensions. As shown in the Figure S2, the distributions of the main set and testing set 2 are largely overlapping, although there are slight differences between the two sets.


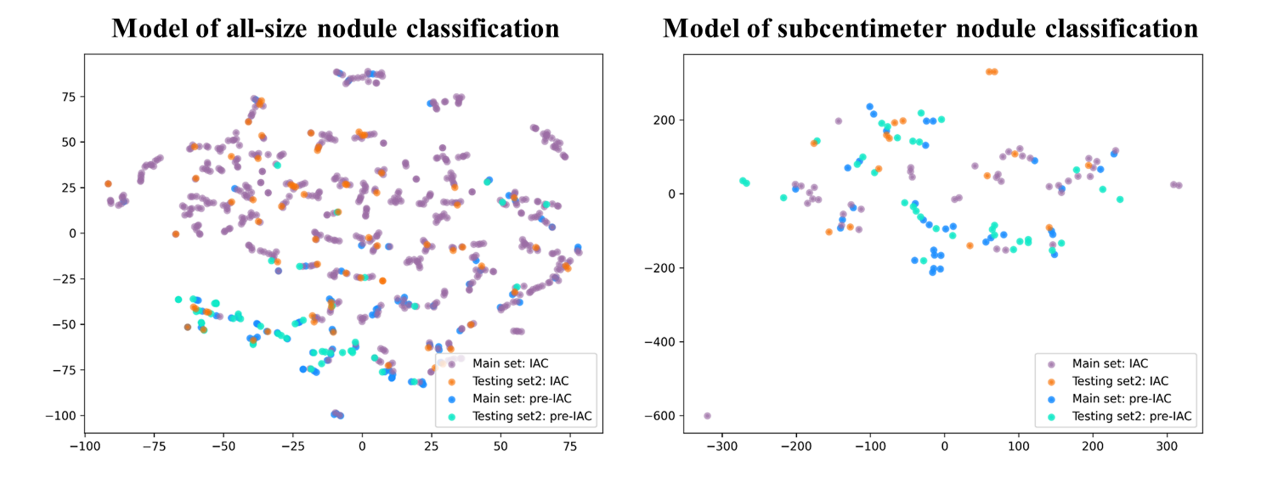


**Figure S2.** Visualization of the deep learning features extracted from the main set and testing set 2. The left corresponds to the all-size nodule classification model, while the right corresponds to the subcentimeter nodule classification model.

Reference

1. Laurens VDM, Hinton G: Visualizing Data using t-SNE. Journal of Machine Learning Research 9:2579-2605, 2008
